# AI for diagnosing malocclusions from 3D dental models

**DOI:** 10.6026/973206300214194

**Published:** 2025-11-15

**Authors:** Imad Mohammed, Subash Chandra Nayak, Vasim Akram Shaik, Akshaya Raj, Murshida Pulayakalathil, Sreejith Karattuparambil Karunakaran

**Affiliations:** 1 Department of Orthodontics, Cmai Medical Center, Uthman bin afan road, Al falah Riyadh, Saudi Arabia; 2 Department of Hi- tech Dental College and Hospital, Odisha, India; 3 Department of Orthodontist, Orthodontic Navodaya Dental College, Raichur, Karnataka, India; 4 Department of Educare Institute of Dental Sciences, Malappuram, Kerala, India; 5 Department of Prosthodontist, Divya Jyoti College of Dental Sciences and Research, Modinagar, Ghaziabad, Uttar Pradesh, India 201204

**Keywords:** Malocclusion, deep learning, orthodontic diagnosis, digital impressions, artificial intelligence

## Abstract

Accurate diagnosis of dental malocclusions remains challenging due to interobserver variability among orthodontists. Therefore, it is of interest to evaluate the diagnostic reliability of artificial intelligence (AI) algorithms in classifying malocclusion types using 3D dental models compared with expert orthodontist assessments. A convolutional neural network (CNN) was trained and tested on digital impressions, and its performance was statistically analyzed against expert diagnoses. Results demonstrated strong agreement between AI predictions and orthodontist evaluations with clinically relevant consistency. These findings highlight the potential of AI-assisted diagnostics to enhance accuracy and reduce subjectivity in orthodontic assessment.

## Background:

Malocclusion is a common dentofacial condition that can impact both esthetics and function, requiring timely diagnosis and treatment [1]. Traditional diagnoses of malocclusion are based on clinical examination, radiographs and dental casts, which can be subjective and time-consuming [2]. The recent introduction of 3D intraoral scanning technology has made it possible to create accurate digital representations of dental arches providing a basis for artificial intelligence (AI) applications in malocclusion diagnosis or treatment planning [3]. Artificial intelligence, often through deep learning networks such as convolutional neural networks (CNNs), has shown promise in the fields of medical and dental diagnosis [4]. Research on the application of AI in orthodontics has considered automated landmark identification in the diagnosis of malocclusion, treatment planning and anomaly identification [5]. Research has shown that CNNs can be trained to classify malocclusion based on the Angle classification system utilizing photographic or radiographic information [6]. However, the assessment of AI models with 3D dental impressions as inputs has yet to be fully explored, particularly in comparative approaches that incorporate comparisons to a benchmark of orthodontists [7]. Comparison studies are essential for robustness and transparency in AI tools for clinical use, as well as notations of their limitations within a naturalistic environment [8]. This study compared the ability of a CNN-based neural network algorithm with that of expert orthodontists to classify Class I, II and III malocclusions from 3D models [9]. Therefore, it is of interest to report the performance of a CNN-based neural network in comparison with expert orthodontists for the classification of Class I, II, and III malocclusions from 3D models.

## Material and Methods:

In this case-control study we used 450 anonymized 3D digital dental models made from TRIOS intraoral scanning. The orthodontic classification (Class I, II, III) was made by three orthodontists with >10 years of clinical experience. A CNN model based on a ResNet-50 architecture was developed using TensorFlow and trained on 300 samples, with 150 reserved for validation. Statistical metrics including accuracy, sensitivity, specificity, Cohen's kappa coefficient and ROC curves were used to evaluate agreement between AI predictions and orthodontist consensus.

## Results:

The CNN model achieved an overall diagnostic accuracy of 92.1% across all malocclusion types. Performance was strongest for Class I, with sensitivity of 94.2%, specificity of 96.3%, and an AUC of 0.95. Class II malocclusion was identified with 89.1% sensitivity, 91.4% specificity, and an AUC of 0.91, while Class III demonstrated slightly lower sensitivity (87.3%) but maintained high specificity (92.2%) and an AUC of 0.90. Agreement analysis confirmed excellent reliability, with a Cohen's kappa of 0.86 between AI predictions and orthodontist consensus, closely comparable to orthodontist inter-rater reliability (0.89). Overall diagnostic agreement between AI and orthodontists was 92.1%, which was nearly identical to inter-orthodontist agreement rates of 93.2% and 93.8%. The dataset distribution showed Class I cases as most prevalent (44.4%), followed by Class II (37.8%) and Class III (17.8%), providing balanced representation across categories, though fewer Class III cases may have contributed to slightly reduced sensitivity. A total of 11 misclassification errors were observed, with most errors involving transitional occlusions. Specifically, 5 Class II cases were misclassified as Class I, 4 Class III as Class II, and 2 Class I as Class III. These findings demonstrate that the AI system delivers near-expert diagnostic performance, particularly excelling in clear-cut cases, while borderline malocclusions remain its primary challenge. The CNN model achieved high diagnostic accuracy across all malocclusion classes, with Class I showing the strongest performance (sensitivity 94.2%, specificity 96.3%, AUC 0.95). Class II malocclusion was classified with 89.1% sensitivity, 91.4% specificity, and an AUC of 0.91. Class III demonstrated slightly lower sensitivity (87.3%) but maintained high specificity (92.2%) with an AUC of 0.90. These results indicate robust diagnostic performance, particularly for Class I. Agreement analysis demonstrated excellent reliability. The Cohen's kappa coefficient between AI predictions and orthodontist consensus was 0.86, reflecting near-perfect agreement, while inter-rater reliability among orthodontists was similarly high at 0.89. Overall diagnostic agreement was 92.1% for AI versus orthodontists, closely matching inter-orthodontist agreement levels of 93.2% and 93.8%. The dataset consisted predominantly of Class I cases (200 cases; 44.4%), followed by Class II (170 cases; 37.8%) and Class III (80 cases; 17.8%). This balanced distribution allowed adequate training and validation across all malocclusion categories, though the smaller representation of Class III may have influenced slightly lower sensitivity. A total of 11 misclassification errors were identified in the validation set. The majority occurred in borderline cases, with 5 Class II cases misclassified as Class I and 4 Class III cases misclassified as Class II. Additionally, 2 Class I cases were misclassified as Class III. Most errors were observed in transitional occlusions, highlighting a diagnostic challenge for the AI in borderline classifications.

Table 1 (see PDF) shows the sensitivity, specificity, and AUC values of the AI model across different malocclusion types, namely Class I, Class II, and Class III. Table 2 (see PDF) depicts the level of agreement measured by kappa values and overall percentage agreement between the AI model and orthodontists, as well as inter-rater reliability among orthodontists. Table 3 (see PDF) presents the distribution of cases across the three malocclusion types (Class I, II, and III), along with their corresponding proportions within the dataset. Table 4 (see PDF) compares the true class and predicted class of malocclusions, highlighting the number of misclassification errors made by the AI model for each category.

## Discussion:

The results of this study demonstrate the clinical potential of AI algorithms to perform accurate malocclusion classification using digital 3D dental models [10]. The CNN model showed strong diagnostic performance, with a kappa value (0.86) that closely approximated inter-orthodontist agreement levels (0.88-0.89) [11]. Comparative literature supports the feasibility of AI in orthodontics which demonstrated similar results using 2D photographic data, reporting 89% diagnostic accuracy for CNN-based malocclusion detection. Our model's ability to handle volumetric input offers a more clinically relevant application for digital workflow environments [12]. Misclassifications in our model, particularly among borderline Class II cases, where transitional occlusion types posed challenges even for human experts [13]. The ROC AUC values above 0.90 across all classes indicate a robust classifier capable of distinguishing nuanced morphological features [14]. From a clinical standpoint, the integration of AI into routine orthodontic diagnostics could reduce inter-clinician variability and expedite patient assessments, especially in teledentistry settings [15]. Ethical considerations remain paramount, with AI intended to support-not replace-clinical judgment. Limitations of this study include the retrospective dataset and lack of radiographic or cephalometric data integration. Future research should evaluate model performance in real-time clinical scenarios, include skeletal data and assess AI's potential in treatment planning.

## Conclusion:

AI models show comparable diagnostic accuracy to orthodontists in classifying malocclusion types using 3D dental models. Integration into clinical diagnostics is promising but requires further validation.
